# The Fate of Heavy Metals and Risk Assessment of Heavy Metal in Pyrolysis Coupling with Acid Washing Treatment for Sewage Sludge

**DOI:** 10.3390/toxics11050447

**Published:** 2023-05-09

**Authors:** Zhiwei Li, Di Yu, Xuejiao Liu, Yin Wang

**Affiliations:** 1 CAS Key Laboratory of Urban Pollutant Conversion, Institute of Urban Environment, Chinese Academy of Sciences, Xiamen 361021, China; zwli@iue.ac.cn (Z.L.);; 2 University of Chinese Academy of Sciences, Beijing 100049, China; 3 Ningbo (Beilun) Zhongke Haixi Industrial Technology Innovation Center, Ningbo 315000, China; 4 Key Laboratory of Urban Environment and Health, Ningbo Observation and Research Station, Institute of Urban Environment, Chinese Academy of Sciences, Xiamen 361021, China

**Keywords:** sludge, pyrolysis, phosphoric acid, heavy metals, removal efficiency, environmental risk

## Abstract

Pyrolysis is an emerging and effective means for sludge disposal. Biochar derived from sludge has broad application prospects, however, is limited by heavy metals. In this study, the fate of heavy metals (HMs) in pyrolysis coupling with acid washing treatment for sewage sludge was comprehensively investigated for the first time. Most of the HMs redistributed in the pyrolyzed residues (biochar) after pyrolysis, and the enrichment order of the HMs was: Zn > Cu > Ni > Cr. Compared with various washing agents, phosphoric acid presented a superior washing effect on most heavy metals (Cu, Zn, and Cr) in biochars derived at low pyrolysis temperature and Ni in biochars derived at high pyrolysis temperature. The optimal washing conditions for heavy metals (including Cu, Zn, Cr, and Ni) removal by H_3_PO_4_ were obtained by batch washing experiments and the response surface methodology (RSM). The total maximum HM removal efficiency was 95.05% under the optimal washing specifications by H_3_PO_4_ (acid concentration of 2.47 mol/L, L/S of 9.85 mL/g, and a washing temperature of 71.18 °C). Kinetic results indicated that the washing process of heavy metals in sludge and biochars was controlled by a mixture of diffusion and surface chemical reactions. After phosphoric acid washing, the leaching concentrations of HMs in the solid residue were further reduced compared with that of biochar, which were below the USEPA limit value (5 mg/L). The solid residue after pyrolysis coupling with acid washing resulted in a low environmental risk for resource utilization (the values of the potential ecological risk index were lower than 20). This work provides an environmentally friendly alternative of pyrolysis coupling with acid washing treatment for sewage sludge from the viewpoint of the utilization of solid waste.

## 1. Introduction

The amount and annual generation of sewage sludge (SS) have skyrocketed with the rapid development of the global urbanization and industrialization in recent years. In China, the amount of SS exceeded 50 million tons in 2020 and may reach 80 million tons by 2025 [1]. SS has been regarded as a bio-resource due to its contents of organic matter and nutrients (N, P, K). Meanwhile, there are numerous forms of harmful organic matter (e.g., pathogens, bacteria carrying antibiotic resistance genes (ARGs) [2], hazardous organic micro-pollutants, aromatic amines and polycyclic aromatic hydrocarbons (PAH)) and high levels of heavy metals (HMs) in SS, which have a huge potential risk to the environment [3,4]. Therefore, it has become an important global concern to develop an environmentally friendly approach for the disposal and utilization of SS.

The conventional SS disposal technologies, including ocean dumping, sanitary landfills, have been restricted in many countries, owing to the poisonous leachate and the waste of land resources [5]. Over the past decade, thermochemical disposal has obtained widespread attention gradually. As one of the most promising thermochemical technologies for SS treatment, pyrolysis possesses several advantages, including nutrient recycling, energy recovery, and high-quality biochar production [3]. Pyrolysis technology for SS treatment can decompose pathogenic bacteria and organic pollutants in solid waste, and reduce the risk of resistance genes [6]. At the same time, sludge pyrolysis produces bio-oil and syngas, which forms a solid by-product, namely biochar, [7]. In the following description, sludge pyrolysis mainly focuses on the solid phase product of pyrolysis, namely sludge-derived biochar. Biochar, a carbon-rich porous material, has aromatic hydrocarbon surfaces, high pH, great cation exchange capacity and nutrient retention capacity [8]. Biochar derived from sludge, owing to its characteristics, has the potential to remediate contaminated soil, improve soil productivity and mitigate climate change [9,10]. Simultaneously, it is a well-established fact that several heavy metals in sludge are accumulated in biochar through the pyrolysis process [11] and the environmental risk of HMs-enriched biochar has attracted much attention [12]. Numerous researchers have dealt with the transformation and speciation evolution of HMs during the SS pyrolysis process [13,14,15]. The HMs in SS could transform from the exchangeable and reducible fractions (bioavailable) to more stable fractions (biounavailable) during pyrolysis, which may be a benefit as it decreases the environmental risk in subsequent application [16]. However, the HMs in SS-derived biochar during the special application may be released when the change in speciation of HMs occurrs in the long-term complex environment [17,18]. Therefore, in order to use sludge-derived biochar more safely, it is necessary to adopt feasible solutions for the removal of heavy metals.

Currently, several methods for heavy metal removal are utilized to reduce potential ecological risks of HMs, including bioleaching, electrokinetic separation, and chemical washing [19]. Chemical washing is a well-established technology with advantages of simplicity, celerity and efficiency, which can be applied to clean HMs contaminated soil [20] and sludge [21]. Generally, common washing agents included inorganic acids, organic acids, and surfactants, among which inorganic acids were the most commonly used [22]. In previous study, the average removal efficiency of Cr, Cd and Pb were 86.9%, 71.3% and 77.2%, respectively, when the sludge was treated by the combination of nitrous acid and nitric acid [23]. The most efficient removal of HMs was obtained by 35% H_3_PO_4_ for the corresponding metal [24]. However, as we know, studies on the quantitative removal of HMs from biochar derived from sludge remain scarce.

Meanwhile, washing agent species, concentration of washing agents, solution pH, S/L(solid liquid ratio) and washing time were reported in previous studies as key factors for heavy metal removal [25]. Therefore, the optimization of washing conditions is particularly necessary. However, the current studies mainly utilized single factor experimental design [19,26], and few studies have been performed on the relationship among these factors for the optimization in the removal of some HMs. To fill in this gap, response surface methodology (RSM) was applied to design washing experiments to evaluate the influence of these influential factors. Meanwhile, some previous studies focused on the improvement of the removal rate of HMs [26,27], while ignoring the speciation distribution and environmental risk assessment of the solid residue after the washing process.

On this basis, the objective of this study is to identify the fate of HMs in pyrolysis coupling with the washing treatment of sewage sludge for the first time. The evolution of HMs in sludge during the pyrolysis process was studied. Furthermore, the acid washing process conditions of sludge pyrolysis residue were optimized and the relationship between various factors was evaluated. The speciation and environmental risks of HMs in solid-phase products after pyrolysis coupling with acid washing were assessed and the mechanism of the removal of HMs was revealed. All these results are believed to deepen understanding of the fate of HMs in sludge in the process of pyrolysis coupled with acid washing and facilitate the sludge-derived biochar application in the field of waste management.

## 2. Materials and Methods

### 2.1. Materials and Reagents

SS sample which had been dehydrated by a hydrothermal treatment coupling plate-frame pressure filtration was collected from a wastewater treatment plant in Xiamen, China. The sludge was in the shape of a hard cake and the general characteristics of SS were investigated, shown in Table 1. The sludge was dried in a drying oven at 105 °C for 24 h until constant weight, and then sieved to a particle size of <125 μm. The reagents used in the experiment are supplied by Sinopharm Chemical Reagent. All these reagents are of analytical purity.

### 2.2. Pyrolysis Experiments

The pyrolysis experiment was conducted in a fixed-bed apparatus under nitrogen flow. The steps are similar to the previous experimental study [1]. According to the pyrolysis temperature x, the sludge-based biochar was named BC-X (Temperature).

### 2.3. Analytical Methods

Thermogravimetric (TG) analysis was performed using a TG analyzer (Netzsch TG 209, Selb, Germany), and the sludge sample was heated from room temperature to 1000 °C under nitrogen flow and a heating rate set as 10 °C/min. The contents of C, H, N, S and O elements of the sample were determined by an elemental analyzer (Vario MAX, Hanau, Germany). To determine the heavy metals in SS and BCs obtained from pyrolysis, the mixed acids (HNO_3_:HF:HClO_4_ = 5:5:2, *v/v*, total 12 mL) in three replicates were injected into the PTFE digestion tube. The specific information about the digestion program steps is in the supplement material. The total concentration of the HMs was determined by the detection of inductively coupled plasma mass spectrometry (ICP-MS) (Agilent 7500 CX, Santa Clara, CA, USA) for the filtrate after the digestion was completed. Error bars represent standard deviations (n = 3).

### 2.4. Acid Washing Experiments

To explore the influence of different washing agents on HMs extraction in the SS and BCs, the samples were washed by various washing agents for 4 h. Washing experiments were conducted in the conical flasks. The vessels were shaken at 200 r/min in a constant temperature shaker. Typically, a weighed amount of SS and BCs was dispersed into 80 mL of washing solution and the range of liquid to solid ratios was from 2 to 12 mL/g. The washing experiments were performed under different acid concentrations (0.05, 0.2, 1.0, 5.0 M), different contact times (from 10 min to 24 h) and three different washing temperatures (25, 50 and 75 °C) in order to determine the effect of different washing conditions for the HMs concentrations in the leachate. After washing, the mixture was centrifuged at 10,000 rpm for 10 min and filtered using a 0.22 μm hydrophilic filter. The HM content of the leachate was determined by ICP-OES. The collected solid was washed three times with deionized water and then dried at 85 °C for 24 h, collected for subsequent determination. All the tests were performed in triplicate and the average values and margin of error were reported for data analysis. The results were presented in this study as the average value and the error was calculated based on the standard deviation formula in Excel.

### 2.5. Optimization Procedure Experimental Design

Based on Box–Behnken design (BBD), the HMs removal washing process was further optimized by response surface methodology (RSM). The level and range of the essential factors (H_3_PO_4_ concentration, washing temperature, and L/S ratio) influencing the washing efficiency were shown in Table S1. In the optimization experiments, the total removal rate of HMs was considered as the response variable. Design Expert software (Version 8.0.5) was introduced to fit the relationship between the efficiency of HM removal and the washing parameter. A quadratic equation model was used to predict the optimal value and elucidate the interaction between the washing parameter as Equation (1):

(1) $$Y=\sum_{i=1}^{n}\alpha_{i}X_{i}+\;\sum_{i=1}^{n}\alpha_{\mathrm{ii}}X_{i}^{2}+\sum_{i=1}^{n}\alpha_{\mathrm{ij}}X_{i}X_{j}{+A}_{0}$$

where Y is the theoretical value; A_0_ is a constant; α_i_, α_ii_, and α_ij_ are the model’s linear, quadratic, and interactive coefficients, respectively; n is the number of variables; and X_i_ and X_j_ are the coded independent variables.

### 2.6. Leachability (TCLP) and Distribution of Heavy Metals (BCR)

The leachability of HMs in the sample was determined by the toxicity characterization (TCLP, US EPA Method 1311) [28]. The chemical speciation of the HMs samples before and after the washing treatment were determined by BCR (Bureau Community of Reference). Each of the four fractions was defined as: F1(exchangeable fraction), F2 (reducible fraction), F3 (oxidizable fraction) and F4 (residual fraction). The specific steps were described in the previous study [29].

### 2.7. Environmental Impact of Heavy Metals

The potential ecological risk index (RI) was usually used to assess the environmental impact of the HMs, which has been adopted by most researchers [5]. The actual calculation program uses the following equations:

C_f_ = C_1_ + C_2_ + C_3_/C_4_ (2)

E_r_ = T_r_ × C_f_ (3)

RI = ∑E_r_ (4)

where C_f_ represents the contamination factor of an individual HM; C_1_, C_2_, C_3_ and C_4_ are the concentrations of the four fractions of the HMs, respectively; E_r_ is the potential ecological risk factor for each HM; and Tr values were the toxic factor of each HM, for Cr, Ni, Cu, Zn, Cd, and Pb were 2, 6, 5, 1, 30, and 5, respectively [5]. The RI values identified four types of risk levels, where RI ≤ 50 represents “low” risk, 50 < RI ≤ 100 represents “moderate” risk, 100 < RI ≤ 200 represents “considerable” risk, and RI > 200 represents “high” risk.

## 3. Results and Discussion

### 3.1. Properties of SS and BCs

The general characteristics of SS and its biochars at different temperatures were investigated, shown in Table 1. The moisture, ash, volatile and fixed carbon contents in SS were 40.28%, 63.74%, 35.07% and 1.19%, respectively. The contents of C, H, N, S and O were 15.44%, 3.25%, 1.62%, 0.55% and 15.4%, respectively. With the increase of pyrolysis temperature, the yield of biochar decreased from 74.64% of the original feedstock mass at 400 °C, 69.86% at 600 °C, to 61.16% at 800 °C. The volatile matter decreased from 35.07% for the raw sludge, 14.65% for the biochar prepared at 400 °C to 1.6% at 800 °C. The ash concentration in the biochar significantly increased while the fixed carbon of all biochar was slightly reduced, which was consistent with other researchers [5,30]. The decrease of the C, H and N content in all the biochar samples was observed from 400 to 800 °C, which was due to the increased volatilization containing these elements during pyrolysis. In addition, The H/C ratio of BCs at different pyrolysis temperatures were all below 2.4, which showed that they were thermochemically converted materials that had a greater proportion of fused aromatic ring structures [18]. The BET surface area (SA) of the biochars was shown in Table 1 as is the increase in biochar surface area with the increasing pyrolysis temperature. For instance, the SA of the BC sample increased by 17.08 m^2^/g more than that of SS when pyrolysis temperature reached 400 °C, whereas the SA of BC800 increased to 49.68 m^2^/g.

To explore the influence of temperature on the thermal decomposition of the sludge, TG analysis was performed in a N_2_ atmosphere. The mass loss as a function of the pyrolysis temperature is depicted in Figure S2. The mass losses for sludge were divided into the following four stages: (1) the first stage was the volatilization stage from 105 to 480 °C, where the total weight loss of the sludge was 22.48%, and the weight loss reached the maximum weight loss rate at approximately 342.42 °C. The DTG peak was not single-peaked, indicating that the pyrolysis in this stage included the superposition of a series of thermal decomposition reactions. This stage mainly included the volatilization and decomposition of fats, carbohydrates and other organic compounds in SS [29]. (2) The pyrolysis rate in the 480–640 °C range was significantly lower; the TG curve tended to be flat, and the weight loss was only 6.32%. It was thus inferred that the main pyrolysis reactions in the previous temperature range were complete. (3) The weight loss rate in this stage was relatively fast in the range of 640 to 825 °C, and the total weight loss in the decomposition stage of the intermediate product was 10.64%, where the maximum weight loss rate occurred at 800 °C from the DTG results, which was probably because the organic matter underwent decomposition, condensation, dehydrogenation, cyclization, etc., and simultaneously, some inorganic substances were thermally decomposed. (4) In the range of 825–1000 °C the weight loss was approximately 7.51%. According to DTG results, the maximum weight loss rate occurred at 925 °C. This stage was the decomposition stage for minerals, including carbonates, alkali metal oxides and chlorides [30]. The undecomposed substances were mainly ash and fixed carbon.

### 3.2. Analysis of HMs

#### 3.2.1. Total Concentration of Heavy Metals

**Figure 1:**
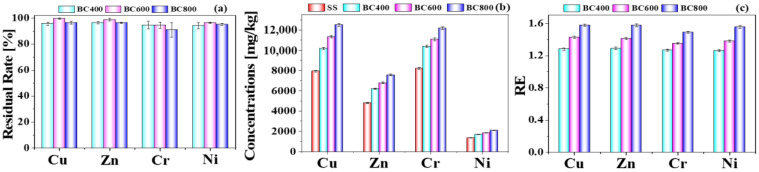
The residual rate (**a**), concentrations (**b**), and relative enrichment factor (RE, (**c**)) of HMs in biochars.

Heavy metals have always been of concern in the disposal of sludge and the application of its biochar. Therefore, it is necessary to analyze the migration behavior of HMs in sludge pyrolysis. The total concentrations of HMs in SS were listed in Table S1. The HM contents in SS decreased as follows: Cr > Cu > Zn > Ni > Pb > As > Cd. Cr had the highest contents among the HMs in the SS, with a maximum content of 8195 mg/kg, which may be due to industrial grinding factories that may use a large number of chromium-containing reagents around this wastewater treatment plant (WWTP). Cu content was about 7945 mg/kg, due to the sewage pipe network being mainly copper pipe in China. According to the relevant Chinese legal standards, the content of Cu, Zn, Cr and Ni in SS exceeded the maximum permitted limit. Because the contents of As, Pb and Cd in SS and its biochars were far below the limit, the subsequent washing experiments focused on Cu, Zn, Cr and Ni in this study. In Figure 1a, the residual rates of Cu, Zn, Cr, and Ni in the biochars were over 96.41%, 98.74%, 94.58%, and 96.42%, respectively, which meant that most of heavy metals mainly redistributed in the biochars, which was consistent with the previous study [31]. Accordingly, shown in Figure 1b, the total concentrations of Cu, Zn, Cr and Ni in biochars increased due to the higher boiling temperatures of heavy metals, so the loss in weight of organic compounds during pyrolysis was higher than the loss in weight of heavy metals [3].

(5) $$\mathrm{RE}=\frac{c_{\mathrm{biochar}}}{c_{\mathrm{feed}}}$$

In Figure 1c, RE represented the relative enrichment factor of heavy metal elements, which was defined as Formula (5), where c_biochar_ and c_feed_ represented the concentrations of HMs in biochar and raw sludge, respectively. The larger the value of RE is than 1, the higher the enrichment degree of HMs in BCs is, and vice versa. Four kinds of HMs were all enriched at 400–800 °C, and the RE values were all above 1.20. Amongst this, the enrichment of Zn was the most obvious. According to the comprehensive comparison, the enrichment order of HMs in sludge biochar was Zn > Cu > Ni > Cr. 

#### 3.2.2. Speciation of Heavy Metals in the SS and Its Biochars

The bioavailability and ecotoxicity of HMs in SS and biochars are mainly associated with chemical speciation. The acid-extractable state (F1) and reducible state (F2), which are very prone to leaching, can directly enter plants through plant roots. The oxidizable (F3) fraction is related to the potentially bioavailable category. The residual state (F4) was unbioavailable because of its strong stability [28].

**Figure 2:**
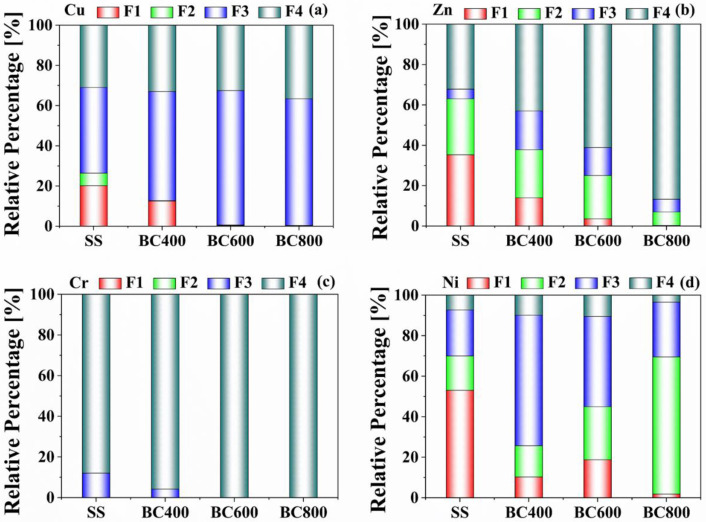
BCR fractions of the HMs (Cu (**a**), Zn (**b**), Cr (**c**), and Ni (**d**)) in SS and its biochars.

Shown in Figure 2, most of the Zn and Ni from SS were primarily distributed in the F1 + F2 fraction (>63%). In comparison, Cu and Cr presented lower environmental risks, as Cu was primarily distributed in the F3 + F4 fraction (73.64%) and Cr remained in the F3 + F4 fraction (>98%). This mainly related to the existence of organometallic or residual phases containing Cu and Cr in raw SS [32]. A remarkable decline of Cu and Zn in F1 and F2 occurred and a significant gradual increase to the F3 + F4 fraction of biochars with increasing pyrolysis temperature from 400–800 °C was presented simultaneously (Figure 2). High pyrolysis temperature also led to the further transformation of Cr from F3 into F4, whereas Cr in BC600 remained in the F4 fraction (99%). The possible immobilization mechanisms were revealed as the decomposition into silicates or metal oxides, the transformation from the amorphous to crystalline state, and the embedding in the C matrix as organometallic compounds [28,33]. Nevertheless, the F3 + F4 fraction of Ni in biochars showed a trend of increasing following decreasing and the F3 + F4 fraction of Ni in BC400 reached the maximum (74.36%). This was probably due to the decomposition of the carbonate state (F1) and the organic bonded state (F3) of Ni in biochar in steps between 400 and 800 °C. In the sequential extraction, the recovery between the sum of the four fractions and the total HM concentration was between 98.65% and 102.47% (Table S2). As a quality control indicator, it showed satisfactory agreement. Although the pyrolysis process had made the HM immobilization to a certain extent, the potential environmental risk still existed due to the high concentrations of HMs which far exceeded the threshold values in the national standards. From the viewpoint of environmental safety to application, further HM washing should be necessary.

### 3.3. Washing Experiments for Heavy Metals Removal

#### 3.3.1. Optimal Washing Agent

**Figure 3:**
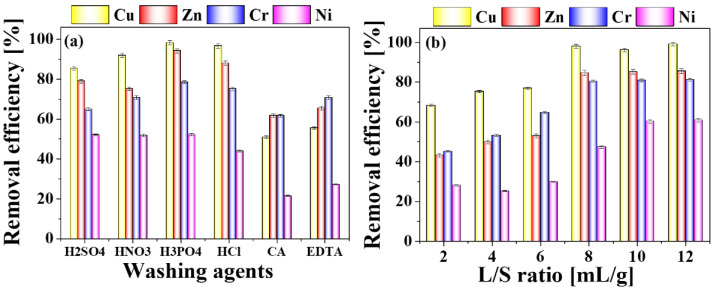
HMs removal efficiencies with different washing agents(**a**) and different L/S ratios (**b**).

To assess the efficiency of different washing agents on the efficiency of HM removal from the sludge-derived biochar, BC400 was extracted by washing agents (H_2_SO_4_, HNO_3_, H_3_PO_4_, HCl, citric acid and EDTA) of 1 M at a L/S of 10 mL/g for 4 h. The findings (Figure 3a) suggested that the removal efficiency of HMs by inorganic acids was higher than organic acid, which were consistent with the report by [34]. In comparison, the removing percentages of Cu (98.26%), Zn (94.31%), Cr (9.11%) and Ni (52.26%) by H_3_PO_4_ were superior to those by the other inorganic acids. The mechanism of the phenomena may be that HCl, H_2_SO_4_ and HNO_3_ in solution can only provide H^+^ and the possible reaction might be:

H^+^ + Me_x_O/Me_x_S/Me_x_CO_3_ → xMe^2/x+^+ H_2_O/H_2_S/CO_2_

The removal efficiency of HMs by H_3_PO_4_ were higher than those by H_2_SO_4_, HNO_3_, and HCl. Differing from the above mechanism, H_3_PO_4_, as a kind of ternary medium-strong acid, provides not only more H^+^ ions, which will react with metal oxide, metal sulfide and metal carbonate, etc. Meanwhile, the PO_4_^3−^ ions, which were produced H^+^ being ionized, also had a larger complexing ability to metal ions [35]. Therefore, the remove efficiency by H_3_PO_4_ is higher than other inorganic acids under the same conditions. These multiple synergisms of H_3_PO_4_ constituted the best removal efficiency results. The possible reactions might be as below:

H^+^ + PO_4_^3−^ + Me_x_O/Me_x_S/Me_x_CO_3_ → [Me(H_2_PO_4_)]^m−^ + [Me(HPO_4_)] ^n−^ + H_2_O/H_2_S/CO_2_

In a subsequent experiment, H_3_PO_4_ was selected for further washing agent of HMs from SS and BCs.

#### 3.3.2. Effect of L/S Ratio on the Removal Efficiency of HMs

The removal efficiency of HMs relates not only to the chemistry of the extractant, as well as the sample geochemistry and characteristics of the metals, but also on dosage of extractants and the process conditions [36]. In Figure 3b, When the L/S ratio was 2 mL/g, the Cu, Zn, Cr and Ni removal efficiencies during H_3_PO_4_ washing were 68.27%, 43.26%, 25.27%, and 28.10%, while the L/S ratio increased to 8 mL/g, the values reached at 98.01%, 84.68%, 80.44%, and 47.44%, respectively. Consequently, the HM removal efficiencies increased with a higher L/S ratio.

#### 3.3.3. Effect of H_3_PO_4_ Concentration on the Removal Efficiency of HMs

**Figure 4:**
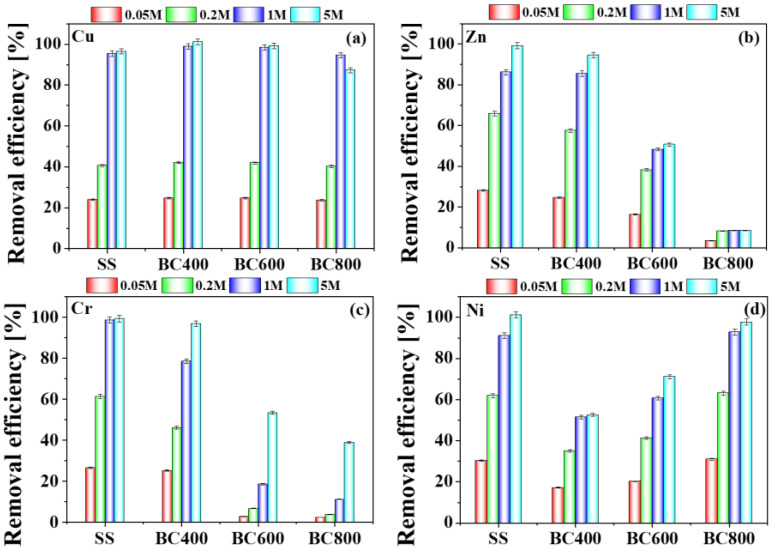
Heavy metals (Cu (**a**), Zn (**b**), Cr (**c**), and Ni (**d**)) removal efficiency from sludge and sludge-derived biochars by H_3_PO_4_ of different concentrations.

The removal efficiencies of Cu, Zn, Cr and Ni in SS and biochars varied with the concentration of H_3_PO_4_ (Figure 4). The removal efficiencies of HMs in SS and biochars significantly increased with the increase of washing agent concentration from 0.05 M to 5 M, which was similar to that reported previously [22]. The removal efficiency of Cu, Zn, and Ni increased sharply from 0.05 M to 1 M. When the H_3_PO_4_ concentration exceeded 1 M, the removal efficiency of Cu, Zn, and Ni changed slightly. When the H_3_PO_4_ concentration was 0.05 M, the removal efficiency of Cu, Zn, and Ni in SS were only 23.85%, 10.96%, and 30.35%, respectively, while those of Cu, Zn, and Ni reached 95.42%, 68.13%, and 93.32%, respectively, when increased to 1 M. However, when the H_3_PO_4_ concentration reached 5 M, the removal of Cu, Zn, and Ni was only increased by 2.67%, 12.57%, and 10.14%, respectively. Meanwhile, it is difficult to filtrate leaching sludge in an excessive concentration of phosphoric acid (5 M). Nevertheless, the removal of Cr was increased with the increasing acid concentration. The removal efficiencies of Cr in SS were 26.57% (0.05 M), 61.40% (0.2 M), 98.60% (1 M) and 99.29 (5 M), respectively. The removal trends of HMs in biochars by different concentrations of H_3_PO_4_ were similar with that of sludge. The effect of pyrolysis temperature for the removal efficiency of HMs was different. Different pyrolysis temperatures have little effect on the removal efficiency of Cu. When the H_3_PO_4_ concentration was 0.2 M, the Cu removal efficiencies of SS, BC400, BC600 were 96.51%, 98.67%, and 99.25%, respectively. Additionally, the removal efficiency of Cu in BC800, decreasing slightly, was about 94.53%. However, the removal efficiency of Zn and Cr in biochars were affected by the pyrolysis temperature greatly. When the H_3_PO_4_ concentration was 5 M, the removal efficiencies of Zn in BC400, BC600 and BC800 were 94.52%, 50.64% and 8.49%, while these of Cr were 96.77%, 53.45%, and 38.89%, respectively. On the contrary, the removal efficiency of Ni in biochars increased from 400 to 800 °C. The removal value of Ni in BC400, BC600, BC800 were 52.57%, 71.68%, and 97.70%, respectively, which corresponded to the BCR result. The above results are mainly related to the different speciation of HMs during sludge pyrolysis. 

#### 3.3.4. Effect of Washing Temperature on the Removal Efficiency of Heavy Metals

**Figure 5:**
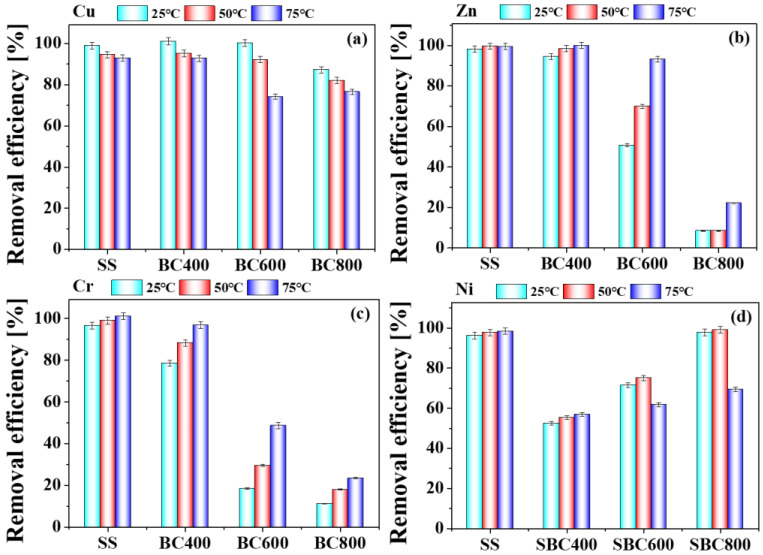
Heavy metals (Cu (**a**), Zn (**b**), Cr (**c**), and Ni (**d**) ) removal efficiency from sludge and sludge derived biochars by H_3_PO_4_ of different washing temperatures (leaching time of 240 min, H_3_PO_4_ concentration of 1 M, and L/S of 8 mL/g).

The effect of washing temperature on the removal efficiencies of HMs in SS and BCs was shown in Figure 5. The removal efficiency of Cr and Zn in sludge and biochars gradually increased with the increase of washing temperature. The washing effect of Zn and Cr at 75 °C increased significantly compared with that at 25 and 50 °C, especially for BC600. The removal efficiencies of Zn and Cr in BC600 increased from 69.90% and 29.55% to 93.21% and 88.62%, respectively, when the leaching temperature increased from 50 °C to 75 °C. Moreover, the removal efficiency of Cu and Ni in biochar with higher pyrolysis temperatures decreased significantly when the washing temperature was 75 °C. Considering the washing effect of various heavy metals, the optimal leaching temperature is about 50 °C.

#### 3.3.5. Effect of Washing Time and the Kinetic Study

**Figure 6:**
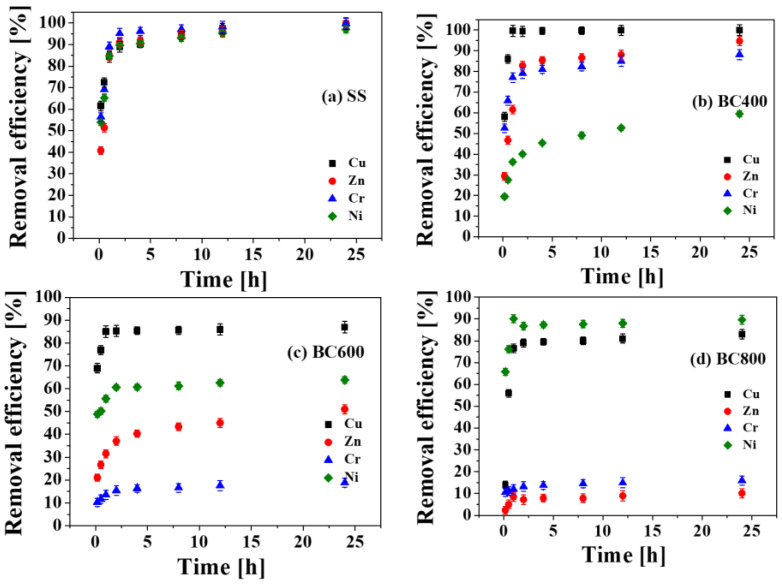
Heavy metals removal efficiency from sludge and sludge derived biochars by H_3_PO_4_ of different washing time (**a**) SS, (**b**) BC400, (**c**) BC600 (**d**) BC800.

Regarding the effect of washing time on the removal of heavy metals in the SS and biochars using 1 M H_3_PO_4_ and a L/S ratio of 1:1 as shown in Figure 6, the concentrations of HMs in the washing effluent increased with time. The removal efficiency of most HMs, except Ni in BC600, presented a rapidly increasing trend then stood in a stable stage, which is consistent with previous studies about HMs leaching from sludge ash [32]. The fundamental molecular theory of liquids was used to explain this result, in other words, the initial acid has the largest hydrogen ion concentration lead to the fastest reaction speed. Subsequently, the hydrogen ion concentration and activity of the acids gradually drop with increasing washing time [37]. The removal efficiency of heavy metals nearly reached equilibrium at about 4 h.

The removal efficiencies of HMs from SS by H_3_PO_4_ first increased, then stabilized, reached at 90.18%, 91.82%, 96.08% and 90.43%, and 99.66%, 85.42%, 80.97% and 45.4%, from BC400. Considering practical application requirements and treatment cost, 4 h was selected for further experiments.

A kinetic study on the removing characteristics of HMs in SS and BCs was performed. The washing process is a heterogeneous reaction process in a liquid-solid reaction system [38]. The shrinking core model (SCM) is the most widely used and is considered to be the kinetic model that best reflects the actual situation of the leaching process [39]. Herein, assuming the residue particle is spherical, the H_3_PO_4_-residue reaction process could be described by the shrinking core model, and was divided into two independent stages, which are controlled by chemical reaction as Equation (6), diffusion of the reagent or product layer as Equation (7), respectively. If the diffusion or the surface chemical reactions are the slowest steps, the equations of the shrinking core models are expressed as follows, respectively:

1 − 2/3 α − (1 − α) ^2/3^ = K_d_ t (6)

1 − (1 − α) ^1/3^ = K_r_ t (7)

However, the R^2^ values of fitting degrees are below 90%, relatively low when using the above equation alone. In order to characterize the kinetic process of the solid-liquid heterogeneous reaction more accurately, based on the shrinking nucleation model of spherical solid particles, segmental fitting of different reaction times was carried out. Table 2 listed the mixed control kinetic model. It is found that R^2^ is greater than 0.95, indicating that the removal of HMs from sludge and biochars is controlled by a mixture of diffusion and surface chemical reactions.

### 3.4. Optimization of the removal of Heavy Metals

The combined effect of washing parameters (including acid concentration, washing temperature, and L/S ratio) on the removal of HMs was further determined by response surface methodology (RSM). The range for the washing specifications were determined from the above single variable experimental results. 17 runs of the total were performed to optimize three variables the values of which are shown in Table S3. A quadratic equation model represented the removal efficiencies for HMs by H_3_PO_4_ as follows: Y = 1.18X_1_ + 1.69X_2_ + 54.56X_3_ − 0.03X_1_X_2_ − 2.04X_1_X_3_ − 0.01X_2_X_3_ + 9.87X_1_^2^ − 0.015X_2_^2^ − 2.86X_3_^2^ − 196.09, where Y represents the HMs removal efficiency; X_1_, X_2_, and X_3_ represent the acid concentration, washing temperature, and washing time, respectively.

**Figure 7:**
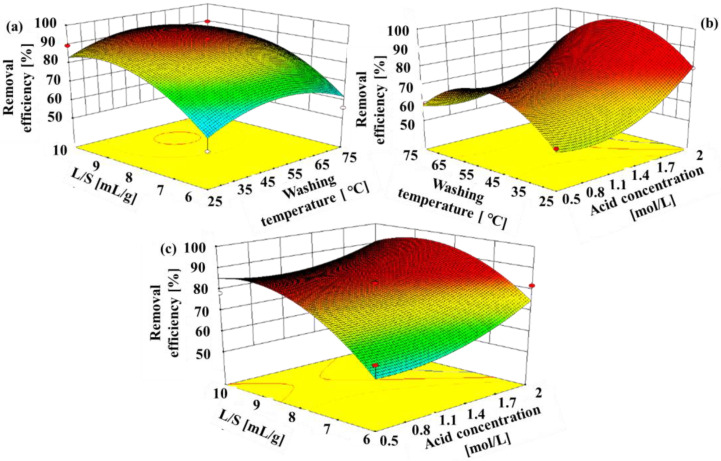
3D plot for the combined effect of experimental factor on the removal efficiency of HMs: (**a**) effects of liquid to L/S ratio and washing temperature, (**b**) effects of H_3_PO_4_ concentration and washing temperature, (**c**) effects of H_3_PO_4_ concentration and L/S ratio.

The extent of the equation fitting was evaluated by ANOVA analysis (Table S4). The F-value was 15.77, which meant that the second-order polynomial equation models was adequate for the optimization study. The *p*-value represents the significance of the influential washing factors and reflect their interaction strength [37]. In this work, the *p*-value of X_1_ was much less than 0.05, which meant that the H_3_PO_4_ concentration(C_0_) was significant in the removal process of HMs. Moreover, the smaller X_1_ and X_2_ *p*-value (*p* = 0.0032) indicated a proper relationship between C_0_ and L/S ratio. In this study, R^2^ (0.9882) and R^2^ (adj.) (0.9768) were close to 1.0, which meant that the significance and agreement between the experimental and predicted values were great. Furthermore, the precision and reliability of the developed models was high because the coefficient of variation value (2.01) was relatively low [40]. In order to evaluate the combined effects of initial concentration, washing temperature and L/S ratio on the removal efficiency of washing by H_3_PO_4_, the 3D mesh diagrams were plotted as shown in Figure 7. According to the response surface analysis, the optimal washing specifications for Cu, Zn, Cr, and Ni removal by H_3_PO_4_ were below: acid concentration of 2.47 M, L/S of 9.85 mL/g, and washing temperature of 71.18 °C. Under these specifications, the predicted maximum HM removal efficiency reached up to 95.61%. Verification experiments were conducted in triplicate at the optimal washing specifications to validate the model suitability. The total HMs removal efficiency was 95.05%, which was in accordance with the predicted values, and the result indicated that the RSM model was accurate for optimizing washing experiment.

### 3.5. Chemical Speciation of Heavy Metals and Environmental Risk Evaluation of SS and BCs after Washing

**Figure 8:**
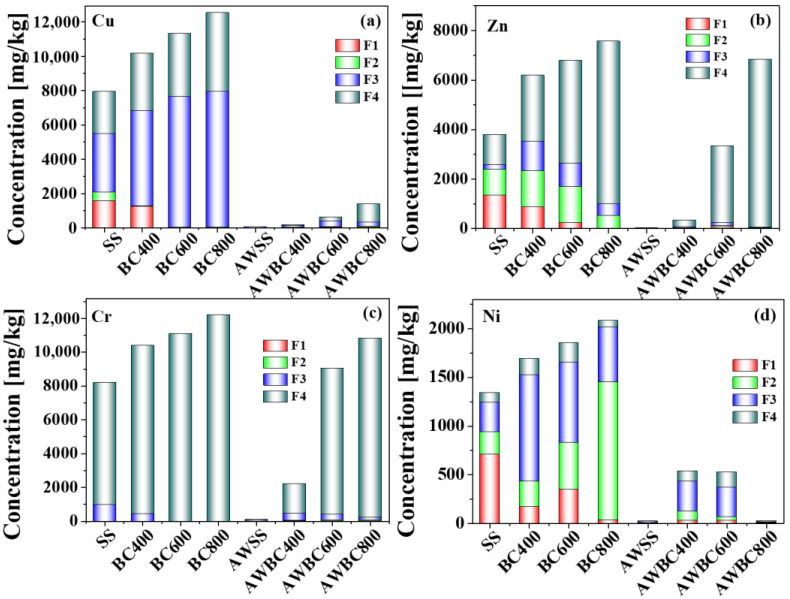
Changes in the fractions of Cu (**a**), Zn (**b**), Cr (**c**), and Ni (**d**) from SS and BCs before and after washing.

The bioavailability and ecotoxicity of HMs in SS and biochars after the washing process are associated with both of the concentration and chemical speciation [30]. The concentration and speciation distribution of HMs in SS and biochars were shown in Figure 8. Compared with the initial HMs distribution in the SS and biochars, the concentrations of HMs in SS and biochars after the washing process were reduced significantly. The concentrations of Cu in SS and BC400 after washing were 73.01 mg/kg and 190.25 mg/kg, respectively, which were both below the control standards for agriculture use (Level A, 500 mg/kg). These in BC600 and BC800 after washing were 645.10 mg/kg and 1450.17 mg/kg, respectively, both below the control standards for agriculture use (Level B, 1500 mg/kg). The concentrations of Zn in SS and BC400 after washing were 30.03 mg/kg and 339.99 mg/kg, respectively, which were below the control standards for agriculture use (Level A, 1500 mg/kg). Furthermore, the one in BC600 after washing was slightly below the control standards for agriculture use (Level B, 3000 mg/kg), about 2980.76 mg/kg. As for the concentrations of Cr in SS and BC400, they were 114.97 mg/kg and 908.12 mg/kg, respectively after washing, which were both below the control standards for agriculture use (Level B, 1000 mg/kg). Moreover, the concentrations of Ni in SS and BC800 after washing were below the control standards for agriculture use (Level B, 200 mg/kg), while the concentrations of Cr in BC800 was 43.99 mg/kg, far below the above standards.

Compared with the HM distribution before washing, the washing process promoted significantly the reduction of the F1 of Cu, Zn and Ni in SS and BCs especially the Zn in SS and BC400, which declined by 91.38% and 68.06%. The F2 fraction Zn in SS, BC600 and BC800 decreased by 96.23%, 94.58% and 97.02%, while that of Ni in BC800 declined by 96.82%. The proportions of F1 and F2 of Cr had little change after washing, less than 1%. In the biochars (BC600, BC800) obtained at higher pyrolysis temperatures, the proportion of F3 decreased while that of F4 increased. The F4 proportions of all four metals in sludge and biochars increased, shown in Figure 6. The loss of more easily extractable fractions and lattice disruption in solid residues was the main reason for the F4 fraction increase among the HMs after washing [41].

To further quantify the environmental effect and potential environmental risk of sludge and BCs after washing (AWSS and AWBC) with H_3_PO_4_, the potential ecological risk index (RI) values of AWSS and AWBCs were assessed in this work. The RI value of SS is about 67.21 (between 50–100), shown in Table 3, indicating a “moderate” risk to the environment. The RI values of BCs, except BC800, were all between 50–100 and were relatively decreased than that of SS, indicating “moderate” risk [29]. However, because the total concentrations of HMs and BCs were large, the leaching risk was still high. After the washing of the solid residue, the RI value decreased significantly to below 50, meaning that the degree of environmental risk was “low”.

The degree of toxicity of HMs in the SS and its biochars is reflected in both the leaching content and the existing speciation of. The leaching concentration of HMs tested by TCLP are listed in Table S5. The leaching concentration of HMs in biochars was significantly lower than that in sludge samples, and the leaching concentration gradually reduced with the increasing pyrolysis temperature. However, HMs (except Cr) in sludge and biochar, especially in low-temperature pyrolytic produce (BC400), exceed the USEPA limits (5 mg/L). After H_3_PO_4_ washing, leaching concentrations of HMs in solid residues were further reduced compared with that of biochar, and all were below the USEPA limit value. From the viewpoint of environmental safety, pyrolysis coupling washing treatment of sewage sludge should be necessary.

## 4. Conclusions

In this work, a novel process for SS treatment through pyrolysis coupled with acid washing was explored and the fate of heavy metals in tandem pyrolysis and washing treatment was comprehensively investigated for the first time. Most heavy metals (HMs) redistributed in the biochars after pyrolysis. The enrichment order of the HMs was: Zn > Cu > Ni > Cr. The phosphoric acid, among various washing agents, had a superior leaching effect on Cu, Zn, and Cr in biochars derived at a low pyrolysis temperature and Ni in biochars derived at a high pyrolysis temperature. According to the optimal results by the response surface methodology, the concentration was more significant for the removal of HMs and a stronger interaction strength between acid concentration and L/S ratio. Further, the total maximum HM removal efficiency reached 95.05%, which was in accordance with the predicted value by RSM, under the optimal conditions (acid concentration of 2.47 M, L/S of 9.85 mL/g, and washing temperature of 71.18 °C). Kinetic results indicated that the washing process was controlled by a mixture of diffusion and surface chemical reactions. After phosphoric acid washing, the leaching concentrations of HMs in the solid residue were further reduced significantly compared with that of biochar, which were below the USEPA limit value (5 mg/L). The solid residue after pyrolysis coupling washing had a low environmental risk for resource utilization (the values of RI were lower than 20). This work provides an alternative of pyrolysis coupling washing treatment for sewage sludge from the viewpoint of environmental safety. Further research would focus on the recovery of heavy metals based on the investigation about fate of HMs in sludge in the process and the evaluation of practical application of pyrolysis coupling washing treatment for sewage sludge.

## Figures and Tables

**Table 1:** General characteristics of SS and its biochars.

Sample	Yield [%]	Ash [%]	Volatiles [%]	Fixed Carbon [%]	C [%]	H [%]	N [%]	S [%]	O ^a^ [%]	H/C [M/M]	S_BET_ [m^2^/g]
SS	--	63.74 ± 0.21	35.07 ± 0.94	1.19 ± 0.73	15.44 ± 0.32	3.25 ± 0.12	1.62 ± 0.06	0.55 ± 0.02	15.4	2.53	17.47
BC400	74.64 ± 0.87	81.44 ± 0.46	14.65 ± 0.87	3.92 ± 0.34	10.4 ± 0.36	2.05 ± 0.14	0.91 ± 0.05	1.09 ± 0.02	4.11	2.37	34.55
BC600	69.86 ± 0.53	86.71 ± 0.12	8.6 ± 0.62	3.7 ± 0.25	9.24 ± 0.57	1.31 ± 0.05	0.49 ± 0.04	1.21 ± 0.04	1.04	1.75	40.51
BC800	61.16 ± 0.77	90.32 ± 0.62	1.6 ± 0.17	2.08 ± 0.46	6.71 ± 0.43	0.87 ± 0.04	0.19 ± 0.03	1.28 ± 0.03	0.63	1.56	49.68

^a^ By difference, O = 100–(C + H + N + S+ Ash).

**Table 2:** Kinetic parameters for the shrinking nucleation model controlled by chemical reaction and diffusion.

Heavy Metals	Sample	Chemical Reaction Control (≤1 h)		Diffusion Control (>1 h)	
		k_r_ (×10^−3^)	Determination Coefficients (R^2^)	k_d_ (×10^−3^)	Determination Coefficients (R^2^)
Cu	SS	3.8	0.9999	10.9	0.9926
	BC400	11.9	0.9998	0.02	0.9427
	BC600	2.9	0.9983	0.008	0.9851
	BC800	6.5	0.9625	0.01	0.9957
Zn	SS	6.2	0.9983	0.1	0.9925
	BC400	3.2	0.9899	0.06	0.9759
	BC600	0.8	0.9781	0.01	0.9817
	BC800	0.4	0.9986	0.005	0.9576
Cr	SS	5.6	0.986	0.06	0.9931
	BC400	3.3	0.9899	0.04	0.9757
	BC600	0.2	0.9993	0.001	0.9644
	BC800	0.1	0.9994	0.007	0.9632
Ni	SS	4.8	0.9851	0.06	0.9821
	BC400	1.4	0.9947	0.02	0.9721
	BC600	0.8	0.9512	0.007	0.9632
	BC800	4.8	0.9946	0.02	0.9869

**Table 3:** Ecological risk assessment of HMs in SS, BCs, ALSS, and ALBCs.

Sample	Cu		Zn		Cr		Ni		RI
	C_f_	E_r_	C_f_	E_r_	C_f_	E_r_	C_f_	E_r_	
SS	2.23	11.14	1.33	1.33	0.14	0.27	9.08	54.47	67.21
BC400	2.03	10.13	1.33	1.33	0.04	0.09	9.08	54.47	66.01
BC600	2.08	10.39	0.64	0.64	0.04	0.08	8.52	51.14	62.24
BC800	1.73	8.66	0.15	0.15	0.00	0.00	28.07	168.45	177.26
ALSS	6.02	30.12	0.08	0.08	1.03	2.06	1.07	6.39	38.66
AWBC400	1.32	6.61	0.23	0.23	0.26	0.53	1.04	6.21	13.58
AWBC600	1.21	6.06	0.08	0.08	0.05	0.10	0.48	2.89	9.11
AWBC800	0.23	1.15	0.01	0.01	0.02	0.04	0.21	1.24	2.44

## Data Availability

Not applicable.

## Supplementary Materials

The following supporting information can be downloaded at: https://www.mdpi.com/article/10.3390/toxics11050447/s1, Figure S1: Schematic diagram of the pyrolysis experimental apparatus; Figure S2: Thermogravimetric curve of chromium-rich tanning sludge; Table S1: Heavy metals concentration of sludge samples and relevant Chinese legal standards; Table S2: The recovery of the sum of four fractions ratio the total HMs concentration; Table S3: The levels of each variable and corresponding heavy metals removal from BC400 obtained from the Box–Behnken design; Table S4: ANOVA for Response Surface Quadratic Model; Table S5: The leaching concentration of heavy metals in sludge and biochars.
