# Supplementary material for: The Fate of Heavy Metals and Risk Assessment of Heavy Metal in Pyrolysis Coupling with Acid Washing Treatment for Sewage Sludge
Source: Toxics. 2023 May 9;11(5):447. doi: 10.3390/toxics11050447 (PMC10224035; doi:10.3390/toxics11050447)
Supplement: Supplementary file 1 [file toxics-11-00447-s001.zip › toxics-2368169-supplementary.pdf]

Supplementary Materials

The Fate of Heavy Metals and Risk Assessment of Heavy Metal in Pyrolysis Coupling with Acid Washing Treatment for Sewage Sludge

Zhiwei Li [a,b], Di Yu [a], Xuejiao Liu [a], Yin Wang [a,c,d]*

a CAS Key Laboratory of Urban Pollutant Conversion, Institute of Urban Environment, Chinese Academy of Sciences, Xiamen 361021, China

b University of Chinese Academy of Sciences, Beijing 100049, China

c Ningbo (Beilun) Zhongke Haixi Industrial Technology Innovation Center, Ningbo 315000, China

d Key Laboratory of Urban Environment and Health, Ningbo Observation and Research Station, Institute of Urban Environment, Chinese Academy of Sciences, Xiamen 361021, Peoples R China

* Corresponding author, E-mail: yinwang@iue.ac.cn

Figure captions

Figure S1: Schematic diagram of the pyrolysis experimental apparatus

Figure S2: Thermogravimetric curve of chromium-rich tanning sludge

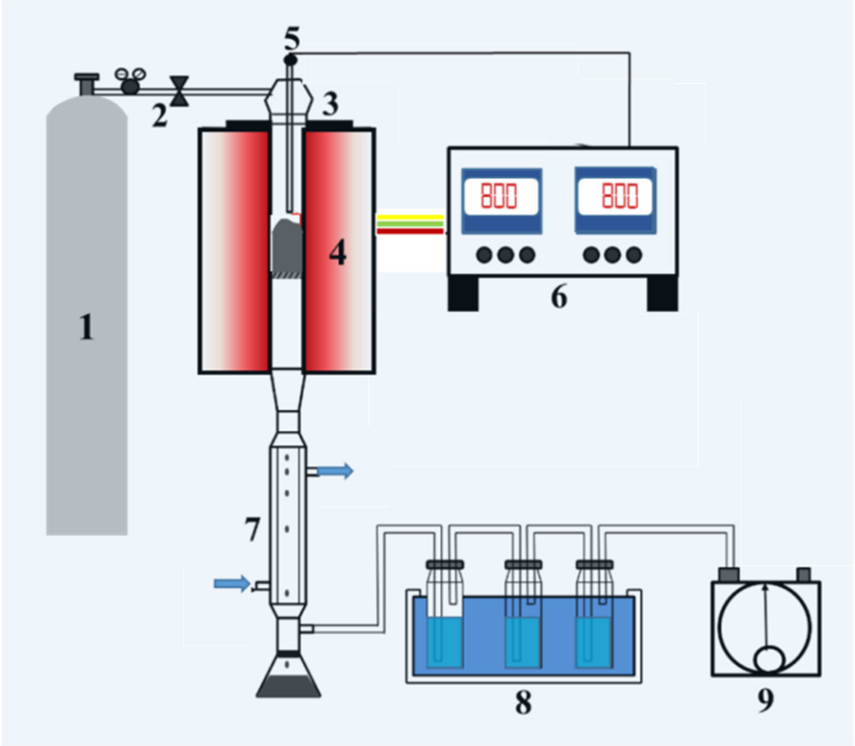

Figure S1. Schematic diagram of the pyrolysis experimental apparatus

(1-Nitrogen; 2-Flow control meter; 3-Fixed bed quartz reactor; 4-Electrically heated furnace; 5-Thermocouple; 6-Temperature controller; 7-Condenser; 8-Acetone trap; 9-Gas collector).

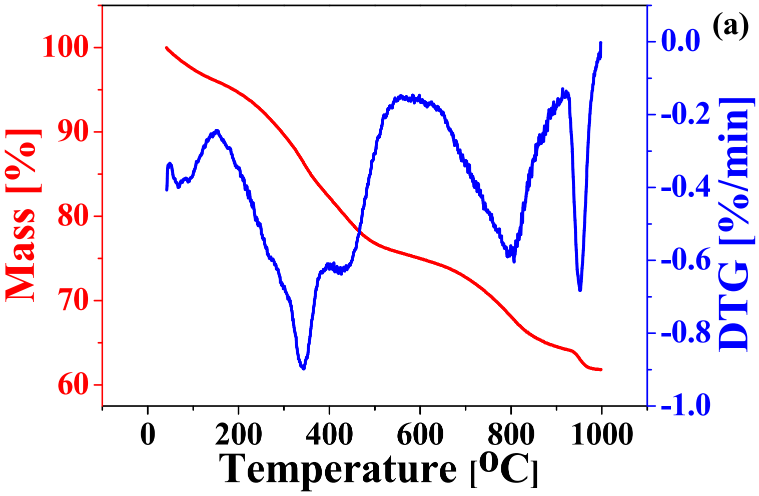

Figure S2 Thermogravimetric curve of chromium-rich tanning sludge.

Table List

Table S1: Heavy metals concentration of sludge samples and relevant Chinese legal standards

Table S2: The recovery of the sum of four fractions ratio the total HMs concentration

Table S3: The levels of each variable and corresponding heavy metals removal from BC400 obtained from the Box–Behnken design

Table S4: ANOVA for Response Surface Quadratic Model

Table S5: The leaching concentration of heavy metals in sludge and biochars

Table S1. Heavy metals concentration of sludge samples and relevant Chinese legal standards.

Heavy metal	Average concentration in sludge (mg/kg)	Standards					
		Quality of sludge used in gardens or parks		Quality of sludge used in land improvement		Control standards for agricultural use	
		pH<6.5	pH>6.5	pH<6.5	pH>6.5	Level A	Level B
Cu	7945.24±30.54	800	1500	800	1500	500	1500
Zn	4794.85±17.32	2000	4000	2000	4000	1500	3000
Cr	8195.13±10.36	600	1000	600	1000	500	1000
Ni	1341.51±4.43	100	200	100	200	100	200
As	24.56±3.64	75	75	75	75	30	75
Pb	29.47±1.03	300	1000	300	1000	300	1000
Cd	1.23±0.06	5	20	5	20	3	15

Table S2. The recovery of the sum of four fractions ratio the total heavy metals concentration.

**Sample**		**Cr concentration of fractions [mg/kg]**				**Sum [mg/kg]**	**Total concentration [mg/kg]**	**Recovery [%]**
		**F1**	**F2**	**F3**	**F4**			
Cu	SS	1640±10.46	506±7.63	3472±5.83	2522±9.73	8140	7945±20.43	102.45
	BC400	1250±9.87	24±1.21	5446±20.65	3317±8.67	10037	10175±19.67	98.64
	BC600	36±2.69	26±0.43	7550±12.67	3664±9.65	11275	11335±33.54	99.47
	BC800	17±1.31	29±0.54	8066±32.75	4681±7.43	12793	12525±37.87	102.14
Zn	SS	1336±7.34	1051±8.93	181±0.72	1220±3.92	3788	3794±21.32	99.83
	BC400	858±6.24	1459±10.66	1180±21.56	2638±8.32	6134	6185±28.22	99.18
	BC600	243±3.15	1461±12.39	950±12.32	4170±10.38	6824	6776±25.87	100.71
	BC800	18±0.23	517±8.93	478±6.32	6640±20.67	7654	7560±24.33	101.24
Cr	SS	0	0	1013±8.23	7377±19.84	8390	8195±29.87	102.38
	BC400	0	0	434±1.64	9936±23.74	10369	10385±30.57	99.85
	BC600	0	0	0	10930±38.90	10930	11080±34.11	98.65
	BC800	0	0	0	12510±34.87	12510	12200±40.05	102.54
Ni	SS	738±1.32	236±0.63	316±0.72	102±1.92	1392	1342±8.71	103.74
	BC400	174±0.84	260±0.84	1089±13.82	168±2.07	1691	1693±3.87	99.86
	BC600	342±1.43	479±1.02	814±11.82	192±1.55	1827	1851±8.54	98.65
	BC800	39±0.32	1446±10.36	578±2.63	73±0.87	2136	2085±10.56	102.47

Table S3. The levels of each variable and corresponding heavy metals removal from BC400 obtained from the Box–Behnken design.

No.	A-H3PO4 Conc. (mol/L)	B:L/S ratio (%)	Washing Temperature (°C)	Removal Efficiency (%)	
				Actual Value	Predicted Value
1	0.5	6	50	71.19	72.71
2	2	6	50	93.18	93.85
3	0.5	10	50	72.64	72.71
4	2	10	50	83.64	80.32
5	0.5	8	25	73.39	73.3
6	2	8	25	75.6	79.01
7	0.5	8	75	70.85	70.18
8	2	8	75	73.75	72.71
9	1.25	6	25	49.28	52.6
10	1.25	10	25	71.57	72.71
11	1.25	6	75	62.15	58.74
12	1.25	10	75	76.62	76.71
13	1.25	8	50	75.69	79.77
14	1.25	8	50	74.4	72.71
15	1.25	8	50	76.4	72.32
16	1.25	8	50	49.73	50.49
17	1.25	8	50	82.36	81.6

Table S4. ANOVA for Response Surface Quadratic Model.

**Source**	**Sum of Squares**	**df**	**Mean Square**	**F Value**	***p*-value Prob > F**	
Model	1788.8	9	198.76	15.77	0.0007	significant
A-A	1090.23	1	1090.23	86.53	< 0.0001	
B-B	3.91	1	3.91	0.31	0.5948	
C-C	66.67	1	66.67	5.29	0.055	
AB	241.96	1	241.96	19.2	0.0032	
AC	8.01	1	8.01	0.64	0.4515	
BC	1.08	1	1.08	0.086	0.778	
A^2	44.99	1	44.99	3.57	0.1007	
B^2	96.86	1	96.86	7.69	0.0276	
C^2	16.53	1	16.53	1.31	0.2897	
Residual	88.2	7	12.6			
Lack of Fit	80.65	3	26.88	14.24	0.0133	significant
Pure Error	7.55	4	1.89			
Cor Total	1877	16				
R^2= 0.9882, R^2(Adj)= 0.9768, C.V.(%)=2.01						

Table S5. The leaching concentration of heavy metals in sludge and biochars.

**Sample**	**Leached concentration (mg/L)**			
	**Cu**	**Zn**	**Cr**	**Ni**
SS	26.41±0.23	25.46±0.32	0.58±0.02	27.58±0.43
BC400	19.82±0.36	17.36±0.25	3.69±0.07	8.82±0.32
BC600	11.91±0.12	1.33±0.05	0.28±0.02	13.14±0.31
BC800	5.92±0.14	0.172±0.02	0.23±0.03	3.02±0.05
AWSS	1.13±0.06	2.32±0.03	0.22±0.01	0.20±0.01
AWBC400	0.17±0.02	1.86±0.03	-	0.88±0.02
AWBC600	1.54±0.03	0.14±0.01	-	0.61±0.03
AWBC800	4.58±0.08	-	-	0.25±0.03
the USEPA limits	5	5	5	5

Note: the SS represents the dry sludge, AWXX represents the sample after washing.
